# Specific increase in T cell potency via structure-based design of a T cell receptor for adoptive immunotherapy

**DOI:** 10.1186/2051-1426-2-S3-P28

**Published:** 2014-11-06

**Authors:** Karolina Malecek, Arsen Grigoryan, Shi Zhong, Wei Jun Gu, Laura A Johnson, Steven A Rosenberg, Timothy Cardozo, Michelle Krogsgaard

**Affiliations:** 1 NYU School of Medicine, New York, NY, United States; 2 Xiangxue Pharmaceutical Co., Ltd, GuangZhou, Peoples Republic of China; 3 NYU, New York, NY, United States; 4 Perelman School of Medicine University of Pennsylvania, Philadelphia, PA, United States; 5 US National Institutes of Health (NIH), Bethesda, MD, United States

## 

Adoptive immunotherapy with antigen-specific T lymphocytes is a powerful strategy for cancer treatment. However, most tumor antigens are non-reactive "self" proteins, which presents an immunotherapy design challenge. Studies have shown that tumor-specific T cell receptors (TCRs) can be transduced into normal peripheral blood lymphocytes, which persist after transfer in about 30% of patients and effectively destroy tumor cells. Still, recent clinical trial with affinity-enhanced TCRs has resulted in severe effects due to cross reactivity to an unrelated peptide. Thus, the challenge for targeted T cell therapy remains to increase T cell potency in order to improve clinical responses and ensure on-target specificity by avoiding unwanted cross reactivity. We used structure-based design to predict point mutations of a TCR (DMF5) that enhance its binding affinity for an agonist tumor differentiation antigen-major histocompatibility complex (pMHC), Mart-1(27L)-HLA-A2, which elicits full T cell activation to trigger immune responses. Structural based approaches have been used to increase TCR affinity, however their potential cross-reactivity has not been reported. Here, we analyzed the effects of selected TCR point mutations alone and in combination on T cell activation potency. Further, we analyzed their specificity and cross-reactivity with related antigens presented by different melanoma cell lines and donor-derived antigen presenting cells. Our structure-based approach allowed us to rationally design sequence substitutions that improve binding in contact areas between the TCR and pMHC without increasing cross-reactivity with a wide variety of self-antigens. We identified and evaluated point mutations in critical TCR positions resulting in more potent T cell activation but maintaining overall specificity. When double and triple combination mutations were introduced, they exhibited an additive enhancement that further improved T cell activation while retaining a high degree of specificity.

## Conclusions

Such affinity-optimized TCRs could potentially be used in adoptive immunotherapy to treat melanoma while minimizing adverse autoimmunity effects.

